# Comparison of Different In Vivo Animal Models of Brachial Plexus Avulsion and Its Application in Pain Study

**DOI:** 10.1155/2020/8875915

**Published:** 2020-11-12

**Authors:** Hang Xian, Rougang Xie, Ceng Luo, Rui Cong

**Affiliations:** ^1^Department of Orthopedics, Xijing Hospital, Fourth Military Medical University, Xi'an 710032, China; ^2^Department of Neurobiology, School of Basic Medicine, Fourth Military Medical University, Xi'an 710032, China

## Abstract

Brachial plexus injuries (BPIs) are high-energy trauma that can result in serious functional problems in the affected upper extremities, and brachial plexus avulsion (BPA) could be considered the most severe type of them. The booming occurrence rate of BPA brings up devastating impact on patients' life. Complications of muscle atrophy, neuropathic pain, and denervation-associated psychological disorders are major challenges in the treatment of BPA. Animal models of BPA are good vehicles for this kind of research. Full understanding of the current in vivo BPA models, which could be classified into anterior approach avulsion, posterior approach avulsion, and closed approach avulsion groups, could help researchers select the appropriate type of models for their studies. Each group of the BPA model has its distinct merits and demerits. An ideal BPA model that can inherit the advantages and make up for the disadvantages is still required for further exploration.

## 1. Introduction

Brachial plexus injury (BPI), which is usually found as the result of posttrauma due to accelerated attacks of the head, neck, and upper limbs, often causes serious functional problems in the affected upper extremities, along with other concomitant injuries to adjacent structures [1, 2]. Most brachial plexus injuries in children that occur during delivery as a result of a traumatic childbirth are known as obstetric brachial plexus palsy (OBPP). The incidence of OBPP ranges from 0.42 to 3 per 1000 live births in Europe as reported [3], while the incidence of adult BPI is still unknown [4]. One retrospective study identified 54 patients of BPI in 4538 (1.2%) patients presenting to a regional trauma facility [5]. The mean age of the patients was 29 years with a male predominance of 89%, while motorcycle and snowmobile accidents were two major causes of adult BPI. Therefore, a distinction between adult versus obstetric BPI should be made for different treatment strategies.

BPI may be described as a traction, rupture, or avulsion injury [6–8]. In a traction injury, the nerve is stretched but not torn from the spinal cord. The degree of injury could vary from neurapraxia and axonotmesis to neurotmesis. In a rupture injury, the nerve is severely stretched and partially or completely torn, which leaves the rupture part outside the spinal column but not the spinal cord, while the attachment of the dorsal root ganglion (DRG) is still intact to the spinal cord (postganglionic lesion) in this type. When the attachment of nerve rootlets is directly torn from the spinal cord, an avulsion injury (preganglionic lesion) happened. This type of avulsion injury is the most serious type of BPI anatomically.

Brachial plexus avulsion (BPA) always requires a tremendous amount of stretching force and is often combined with multiple injuries of the affected limb or others, which is considered the most severe type injury of the upper limb [9]. The occurrence rate of avulsion of one or more roots from the spinal cord, which can be defined as brachial plexus root avulsion, reaches up to approximately 70% in BPA [1]. Because of the concomitant injury to adjacent structures, especially to the spinal cord or principal arteries, prehospital emergency treatment and advanced life support are always needed in the management of this devastating injury. Due to which, early diagnosis of BPA has often been masked. Motor dysfunction and skin anesthesia of the upper limb are the main symptoms of BPA, and total loss of these functions is often observed on the global avulsion patients who are carrying paralyzed upper limbs. As both of peripheral and central nervous systems are involved in this root avulsion injury, BPA may pose its unique characteristics during the whole pathophysiological process.

Muscle atrophy due to chronic denervation and misdirection of regenerating axons, which finally results in poor motor function of the affected limb, is still the main complication of BPA, although much effort has been made in the treatment procedure of nerve reconstruction [10]. The lack of adequate proximal intraplexus donors (roots) in continuity with the spinal cord is still a thorny issue that the reconstructive surgeons must face [11]. Dorsal root avulsion brings up permanent impairment of sensory functions due to disconnection between the peripheral and central nervous system. The frequency of the development of neuropathic pain after BPA is also another great issue that brings a devastating consequence to BPA patients. The features, such as systemic mechanical and thermal hyperalgesia, which appear immediately after the injury and produce long-lasting pain behavior, and the following pathological plasticity of the central and peripheral nervous systems are triggered by a series of cytokine cascades [12–14]. All the reactivity above will finally achieve a stable and long-lasting neuropathic pain, and the BPA patients are more susceptible to such neuropathic pain with an incidence rate of 50% in BPIs up to 90% in BPAs according to epidemic data [8, 15, 16].

Big challenges on the process of BPA treatment including prevention of nerve degeneration, recovery of limb sensation, and management of neuropathic pain still exist, although lots of efforts have been made. To address these issues, several animal models have been established with attempts to simulate the injury mechanisms of BPA, and most of them are useful in the experimental studies of the pathological process of this injury. Animal models have made great contributions to the field of spotting a novel treatment target and characterizing specific medications. Among the current BPA models, different species, including rat, mice, and rabbit, are well designed to mimic the avulsion mechanism and pathological state like those in BPA patients. These models could be classified into three categories according to different surgical approaches, which include the anterior approach avulsion, the posterior approach avulsion, and the closed approach avulsion. It is very difficult for researchers to select a consummate model to match their experiments, so special attention has been paid in this review to the merits and demerits of each approach of the in vivo BPA models.

## 2. The Anatomy of the Brachial Plexus

The basic feature of the brachial plexus is formed by the anterior spinal nerves from the four lowest cervical roots and the first thoracic root despite significant intra- and interindividual variability [17]. After punch out from the intervertebral foramen, these five nerve roots (C5-T1) form the brachial plexus between the scalenus anterior and the scalenus medius muscles. The brachial plexus could be divided into five different segments according to specific anatomy landmarks, which are roots, trunks, divisions, cords, and branches [18]. The spinal roots unite to form three trunks including the upper trunk (C5 and C6), middle trunk (C7), and lower trunk (C8 and T1). Two terminal nerve branches emerge at the root level including the dorsal scapular nerve (C4-5) and the long thoracic nerve (C5-7), which supplies the levator scapulae and rhomboid muscles, and the serratus anterior muscle, respectively. After travelling posterior to the clavicle, each trunk then divides into two divisions including anterior division and posterior division. Two terminal nerve branches emerge at the trunk level including the suprascapular nerve (C5-6) and the nerve to the subclavius muscle (C5-6). The suprascapular nerve arises from the superolateral aspect of the upper trunk, the location of which is referred to as Erb's point. After the brachial plexus has become infraclavicular distal to the clavicle, the anterior divisions of the upper and middle trunks form the lateral cord, while the lower trunk becomes the medial cord. The posterior divisions of every trunk form the posterior cord by contributing different proportions of spinal roots. The anatomic relationship among the cords and the second part of the axillary artery is the reference standard of the naming of them, and all of them are located posterior to the pectoralis minor muscle. Finally, five main terminal branches including radial nerve, axillary nerve, ulnar nerve, musculocutaneous nerve, and median nerve are formed to govern the motor and sensation of the upper limb.

Anatomically speaking, one species of animal, which could be the most suitable one in BPA modeling, should firstly meet the similarity of the anatomical structure with that of a human being. Bogusch [19] has reported that mice have similar brachial plexus anatomy as humans, which is also built up by spinal nerves C5-C8 and T1. Even if there is some anatomical variation, constant nerve root composition of the mouse brachial plexus is adequate for research use. A rat's brachial plexus also has similar anatomic structure as that of humans, which has been verified by researchers [20, 21]. Similar work has been done on rabbits, and the anatomy results are similar to those of mice and rats [22]. Secondly, an easier operation performance seems to be another issue in animal model selection. During the process of mouse modeling, all procedures should be completed under a microscope for higher precision. This kind of difficulty may not exist in bigger rodents like rats or rabbits. Finally, an easy animal acquisition and convenient management are also of great importance in animal selection. Nevertheless, mouse and rat models are still the most popular BPA models in the field of BPA researches, which will be fully reviewed in the latter parts.

## 3. Three Approaches of the Animal Avulsion Modeling Procedure

### 3.1. Anterior Approach Avulsion

#### 3.1.1. Procedure

One representative surgical method of the anterior approach avulsion is from the research of Rodrigues-Filho et al. [23] in 2003, which is a lower trunk (C8-T1) rat BPA model. Briefly, the rat was placed on an operating table in supine position after intraperitoneally injecting anesthesia. A horizontal incision parallel to the clavicle, which ran from the sternum to the axillary region, was made for a clear view of the right brachial plexus. The cephalic vein should be carefully protected after the pectoralis major muscle was displaced. The division and cord part of the brachial plexus, which was wrapped in an axillary sheath together with a brachial artery, could be observed after a deeper anatomy in the anterior margin of the pectoralis minor. Trace to the proximal end till the vertebral foramen where the C5-C8 and T1 branches were exited, and the lower trunk of the brachial plexus was carefully identified and dissected. The lower trunk was grasped with forceps and extracted from the spinal cord by steady moderate traction. Tissue layers were finally brought together, and the skin was sutured. The mark of a successful avulsion is the visibility of the corresponding DRGs of the nerve roots. A similar BPA model procedure adapted for mice was performed by Quintão et al. [24] in their research.

#### 3.1.2. Current Research Focus

On the BPA model exploration part, single lower trunk avulsion may not meet the clinical and scientific needs for further studies. Li et al. [25] reported a global avulsion of the brachial plexus by using a similar method above for the study of motor neuron apoptosis. Thereafter, a novel BPA rat model of the upper trunk was designed by Liu et al. [26], which showed a persistent neuropathic pain behavior in ipsilateral and contralateral limbs, and may be a suitable animal model for neuropathic pain research. One C7 root avulsion by forceps of a rat model was invented to study the neuroprotective effect of minocycline via different routes [27]. On the research exploration part, BPA models are widely used in the study of BPA-induced neuropathic pain. Avulsion of the brachial plexus in a rat produces persistent mechanical and cold allodynia and mechanical hyperalgesia, all of which are the characteristics of neuropathic pain [23, 28]. Quintão et al. [13] found that neurotrophic factor blockers might represent a new and interesting option for the management of neuropathic pain. Paszcuk et al. [29] reported that cannabinoid agonists could inhibit neuropathic pain induced by BPA which could be a new analgesic target. Similar work has been done by Kobayashi et al. [30] on the application of the p75NTR inhibitory antibody and Zhao and Wu [31] on histone deacetylase inhibition to suppress neuropathic pain after BPA. Recent work on level changes of microRNA could supply other new ideas in BPA-induced neuropathic pain treatment [32], and enhancer of zeste homolog 2 (EZH2) was proven to be a novel regulator of neuroinflammation and neuropathic pain via the mTOR signaling pathway in the anterior cingulate cortex, which could be another target of pain treatment [33]. In other words, an anterior approach BPA model of mice or rats at any trunk of the brachial plexus could be implemented and become a good model of related research, especially on the research in the neuropathic pain field.

#### 3.1.3. Merits and Demerits

In this group, the first merit is that the whole anterior procedure could be completed through a smaller incision with little hemorrhage and high security. Second, this approach could get a better view of the total brachial plexus roots, especially the middle and lower trunks, which could help the researchers perform the avulsion procedure conveniently. Then, the force direction of avulsion in this position could well simulate the injury of BPA patients, and whether the avulsion is successful or not could be evaluated by the vision of corresponding DRGs directly during the modeling procedure. Meanwhile, there are also some demerits. Forceps are used to hold the trunk before avulsion, and this kind of clamping force could cause the first step injury to the nerves. Whether this kind of force has an effect on model assessment or not remains inconclusive. In terms of pain behavioral tests, all the tests are performed on other extremities except the avulsion one. Whether this indirect pain response can represent the real pain state of BPA models is still unknown.

**Figure 1:**
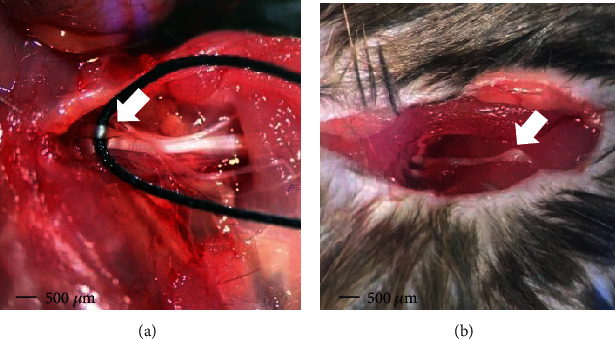
Surgical procedure of the novel C7 single root avulsion mouse model through an anterior approach: (a) a soft silk thread was used to hook the C7 nerve root for the preparation of avulsion injury (white arrow); (b) the avulsed C7 nerve root where the DRG of this root could be observed (white arrow).

To compensate for the shortcomings above, our research group has designed a novel anterior approach BPA model of mice, which is the seventh cervical nerve root (C7) avulsion model. This novel C7 single root avulsion mouse model could not only well mimic the mechanism of the avulsion injury like that in patients but also show a persistent pathological status of neuropathic pain like that in the anterior avulsion models used above. During avulsion operation, we replaced the forceps by a soft silk thread to hook the C7 nerve root and then performed the avulsion, which eliminated the first step injury to the nerves (Figure 1). The behavioral tests including mechanical allodynia and cold hyperalgesia of the ipsilateral fore paw, contralateral fore paw, and ipsilateral hind paw showed that this pain state could last for 28 days after modeling without affecting the grasp force of the fore paw [34]. This direct pain detection and evaluation through tests of the affected fore paw could supply more direct evidence of the neuropathic pain status induced by BPA than other anterior approach models.

### 3.2. Posterior Approach Avulsion

#### 3.2.1. Procedure

Primarily, a posterior approach of BPA is introduced for the research of root reimplantation into the spinal cord for repair of the avulsed brachial plexus nerve roots [21]. The procedure is described briefly as follows. The rat was placed on an operating table in prone position after intraperitoneally injecting anesthesia. A 3-5 cm length skin incision through the dorsal midline of the rat was made, and the paraspinal muscles were segregated with a pair of ophthalmic scissors. The prominent spinous process of the C7 vertebrae was used as a landmark; the hemilaminectomy from C4 to T1 on the right was performed to expose the spinal cord through a posterior surgical approach. After adequate hemostasis, the target roots (like C5-T1) of the brachial plexus is identified and confirmed. Then, target roots were avulsed from the spinal cord with a micronerve hook. A successful avulsion could be directly observed under the naked eyes or microscope. Tissue layers were finally brought together, and the skin was sutured after adequate hemostasis again. The ventral and/or dorsal roots of each spinal nerve could be observed clearly in this posterior approach, so a precise ventral or dorsal root level of avulsion also could be achieved through it.

#### 3.2.2. Current Research Focus

Similar to an anterior approach, there are also different root combination avulsion by this posterior approach, as Muñetón-Gómez et al. [35] reported a rat model of global BPA through a posterior approach which was used to evaluate the regeneration and reparative therapy of BPA. More precise avulsion of rats' C7 root by Sim et al. [36] and C8 root by Gu et al. [37] was used to evaluate the neuroprotective effect of paclitaxel in the prevention of motoneuron death and mitochondrial dysfunction and to investigate the survival, regeneration, and functional recovery of motor neuron by reimplantation of the ventral root method. Another pediatric pig avulsion model of upper and middle trunks through this approach was used to describe the functional and neuropathological outcome following BPA, with or without spinal cord injury [38]. Similar rat or mouse models of upper-middle trunk and global trunk avulsion were successively reported to expound the mechanism of motor neuron degeneration and/or to evaluate different repair and reconstruction methods [39–41]. A most recent research of Huang et al. [42] reported that the temperature-sensitive quercetin-loaded PLGA-PEG-PLGA hydrogel sustained-release system could promote nerve regeneration and motor function recovery during the early stage of BPA due to its intrinsic function of anti-inflammation, which had a good prospect from bench to bedside translation. Meanwhile, the posterior approach models also could be used in the field of neuropathic pain research according to recent work. Hou and Xu [43] reported that the activation of astrocytes and microglia in the spinal cord played a key role in a rat model of global BPA. Meng et al. [44] found that reduced lncRNA Malat1 expression might induce neuropathic pain by increasing neuronal excitability in the spinal cord via regulation of calcium flux, and Huo et al. [45] reported the beneficial effects of electroacupuncture for relieving neuropathic pain in global BPA rats and also explored its function in brain plasticity. It appears that more and more researches on higher central nervous systems based on this kind of BPA models are emerging to further elucidate the networks of sensory information processing [15, 46]. While the search for the improvement of such models have not stopped, Fang et al. [47] invented a new method through using a weight attached to the forceps or hook during avulsion to achieve a better conditional homogeneity and the initial results were preferable. In a word, a posterior approach BPA model of mice or rats appears to be more popular in exploration of the motor neuron-associated mechanisms and reconstruction procedure evaluation.

#### 3.2.3. Merits and Demerits

In this group, the avulsion roots could be definitely confirmed by boney landmarks of cervical vertebrae, and more precise ventral and/or dorsal root avulsion could be easily achieved through this approach. Then, root replantation or other reconstruction operations could be implemented through this approach. Despite the merits above, there are still some demerits. Bigger incision and wound of this approach increase the risk of hemorrhage and cerebrospinal fluid leakage. The hemilaminectomy performed not only can affect the stability of the spinal cord but also can bring up serious complications like paraplegia and death of animals. The avulsion through this approach cannot well simulate the avulsion injury of BPA patients, which may be the reason that studies based on this kind of models are more in the field of motor neuron degeneration and reconstruction compared with the anterior approach ones.

### 3.3. Closed Approach Avulsion

#### 3.3.1. Procedure

In 2000, Spinner et al. [48] established a rat model of BPA using passive acceleration, which could be classified as the closed avulsion approach. In brief, the rat was placed on its right side within a specially designed tube (6 cm in diameter) after intraperitoneally injecting anesthesia. The right forelimb protruded from an oval cutout on the undersurface of the tube. The limb made an approximately vertical angle with the front and back of the torso in this position. Then, the elbow was placed in a designed metal sleeve which was tightly fixed to a lever arm. The force was applied four times in distance from the fulcrum as the sleeve so that the forelimb supported the weight of the lever arm. An instantaneous force was applied to the limb by dropping a precast test weight down the guide rod from a height (about 30 cm) onto the lever to simulate the injury mechanism like that in patients. The animals were sacrificed to confirm the effect of avulsion. A similar close avulsion rat model was established by Yang et al. [49] through using a specially made model making apparatus.

#### 3.3.2. Current Research Focus

Closed avulsion models seem to be the ideal in vivo models of BPA, and they may well simulate the injury mechanism like that in BPA patients. Nevertheless, there is still little research on the mechanism study by using this noninvasive models, and the existing models are confined only to rat species. Further studies are called for on this model with an expectation on producing reliable preclinical evidence in the field of therapeutic strategy exploration and assessment of BPA.

#### 3.3.3. Merits and Demerits

Compared with the invasive group above, this noninvasive approach method could be considered the unique merit of this group. The force pattern that induces the avulsion injury could perfectly mimic the avulsion force like the injury experiences of BPA patients. This closed approach requires a series of avulsion facilities and complicated operations which may bring up a longer learning curve of this technique. Precise avulsion of any specific trunk is difficult to be achieved during modeling, and definite avulsion level of roots or trunks is difficult to be evaluated after modeling, both of which may be the obvious demerits of this avulsion approach.

For the detailed overview of the in vivo models of brachial plexus avulsion, refer to Table 1.

## 4. BPA-Induced Neuropathic Pain and Treatment

As the injury mechanisms of BPA is different from other kinds of BPI, in which both the peripheral nervous system and the central nervous system are directly involved at the initial avulsion, therefore, the complications following BPA should be treated differently. Neuropathic pain often develops after the lesion or disease affecting the somatosensory nervous system [50, 51], which could be of central or peripheral origin depending on the location of the lesion or disease [52]. The main clinical symptoms of neuropathic pain include spontaneous pain, hyperalgesia, and allodynia of the affected limb [53], which does not usually respond to general analgesics or which responds only to a small extent. This kind of chronic pathological pain state often not only represents a challenge to clinical practice and basic science but also brings up devastating impact on patients' life [54]. Pain following avulsion injury of the brachial plexus has been early recognized [55], which is considered the most distressing complications of it. It is reported that the incidence of chronic pain following BPI is as high as 50% [56, 57] and even up to 80% [58], and similar incidence rate was also reported in BPAs [59, 60]. The underlying various physiological and pathological mechanisms of the BPA injury are far to be elucidated which may be the main reason for its intractable management.

Previous studies suggested that this neuropathic pain induced by BPA might be alleviated through surgeries including successful nerve transfer procedures to restore limb functions and dorsal root entry zone (DREZ) lesions. As reported by Berman et al. [61], pain reduction might anticipate functional recovery following nerve transfer strategies for BPA and the underlying mechanism might include successful regeneration and/or restoration of peripheral connections prior to their function, especially in the muscle. BPA models of the posterior approach are widely used in this area to investigate the underlying mechanism of motor neuron degeneration and pain and test the treatment effect of different reconstruction strategies and its profound mechanisms [37, 40, 41]. Multiple evidences of experimental and clinical background suggest that the spinal dorsal horn (SDH) is at least the partial location of the pain generator after BPA occurred due to deafferentation [62, 63], so surgeries targeting the location of SDH, especially the DREZ, are supposed to be effective in the treatment of neuropathic pain after BPA. It is reported that neuropathic pain induced by the dorsal horn deafferentation by cervical posterior rhizotomy was successfully relieved through microsurgical DREZ rhizotomy/coagulation [64]. A long-term follow-up study in patients with deafferentation pain due to BPA showed pain relief gradually decreased over 5 years after surgery [65], and similar satisfactory treatment effect has been achieved in other studies [66, 67]. Other therapeutic attempts through surgical procedures also emerged in recent years, which include motor cortex stimulation, thalamic deep brain stimulation, spinal cord stimulation, and DRG stimulation [68–72], and the treatment effects seem satisfying as far as it goes.

The current analgesic treatment for neuropathic pain according to the guidelines include the calcium channel acting as anticonvulsants pregabalin and gabapentin, tricyclic antidepressants, and serotonin-noradrenalin-reuptake inhibitors (duloxetine, venlafaxine), which are the first-line treatment recommendations [52, 73], while the search for better chemical compounds to treat neuropathic pain is still going on. The analgesic effect of the reported researches is still ambiguous [74], which may be due to the reason that the mechanisms of neuropathic pain are a complex pathophysiological process involving multiple anatomy levels that remains to be elucidated. In vivo BPA models are ideal carriers in the field of treatment target selection and assessment. As reported by Quintão and his colleagues [12], selective B_1_R antagonists might well represent valuable tools for neuropathic pain management. Our previous work has been done on the molecules, including cyclic GMP-dependent protein kinase-I (PKG-1), CCL2/CCR2, and canonical transient receptor potential channels (TRPC) [75–78], which showed the potential on the treatment of neuropathic pain, but whether this kind of molecules shares the same pathophysiological functions in different BPA-induced neuropathic pain still needs further studies. Therapeutic strategies for the treatment of neuropathic pain are always limited by its elusive mechanisms and the incomplete understanding of nervous system plasticity after the initial injury [79], so we still need to search for different targets to treat this pain. Researches targeting the gene level have emerged with the development of genomics, and the results of the preliminary investigations seemed promising based on BPA models [32, 33, 44], which may be another novel route of the BPA treatment.

Personally speaking, I still call for further improvement of the innovation in BPA modeling to inherit the advantages and make up for the disadvantages. In terms of the existing models, studies on mouse models are relatively scarce. Operations on mouse models may require a higher micromanipulation technique, but characteristics of mouse gene polymorphism, which allows us to use genetic tools and to conduct experiments at the gene therapy level, could make up for its shortcomings to a great extent. In terms of neuropathic pain, models that could well simulate the characteristics of BPA-induced neuropathic pain which could be directly detected and evaluated by associated tests on the affected extremity will better help us in understanding its mechanism and finally guide the choice of treatment. Although there is not absolutely the same model, the accurate reproduction of models is also an issue that needs to be seriously considered during new model design.

## 5. Conclusion

Brachial plexus avulsion injuries are defined as devastating life-altering injuries which could cause multiple complications like extremity disabilities, intractable pain, and negative emotions. The underlying elusive mechanisms make it difficult to be cured. Selecting suitable in vivo animal models that share an extremely similar injury mechanism, pathophysiological process, and recovery potential as humans not only can help us clarify the mechanisms better but also can facilitate the translation of basic science to the clinical settings. The current in vivo animal models of BPA could meet the initial requirements of both scientific and clinical researches, while there is still need for more advanced BPA models in the future to best match the needs to help researchers explore the further prognosis of the avulsion injuries and finally benefit the BPA patients.

## Figures and Tables

**Table 1:** Detailed overview of the in vivo models of brachial plexus avulsion.

Surgical approach	Authorship	Publication year	Species	Type of avulsion	Research field	Refs
Anterior avulsion approach	Rodrigues-Filho et al.	2003	Rats	C8-T1 roots	Neuropathic pain	[23]
	Quintão et al.	2008 2006	Mice	C8-T1 roots	Neuropathic pain	[13, 24]
	Li et al.	2015	Rats	C5-T1 roots	SC motor neuron apoptosis	[25]
	Liu et al.	2016	Rats	C5-C6 roots C5-T1 roots	Neuropathic pain	[26]
	Tan et al.	2017	Rats	C7 root	Neuroprotective effect	[27]
	Wang et al.	2015	Rats	C5-T1 roots	Neuropathic pain	[28]
	Paszcuk et al.	2011	Mice	C8-T1 roots	Neuropathic pain	[29]
	Kobayashi et al.	2015	Rats	C8-T1 roots	Neuropathic pain	[30]
	Zhao et al.	2018	Rats	C8-T1 roots	Neuropathic pain	[31]
	Liu et al.	2018	Rats	C5-T1 roots	Neuropathic pain	[32]
	Meng et al.	2020	Rats	C5-T1 roots	Neuropathic pain	[33]
	Zhang et al.	2020	Mice	C7 root	Neuropathic pain	[34]
Posterior avulsion approach	Cao et al.	2003	Rats	N. S.	Anatomic and technical exploration	[21]
	Muñetón-Gómez et al.	2004	Rats	C3-T3 roots	Nerve regeneration	[35]
	Sim et al.	2015	Rats	C7 root	SC motor neuron protection	[36]
	Gu et al.	2004	Rats	C8 root	SC motor neuron regeneration	[37]
	Zarina et al.	2016	Pigs	C5-C7 roots	Neuropathological characteristics	[38]
	Zhang et al.	2017	Rats	C5-T1 roots	Motor cortical reorganization	[39]
	Chen et al.	2019	Mice	C5-C7 roots	SC motor neuron protection	[40]
	Zhang et al.	2019	Rats	C5-C7 roots	SC motor neuron protection and reconstruction methods	[41]
	Huang et al.	2020	Rats	C5-C7 roots	Nerve regeneration	[42]
	Hou et al.	2018	Rats	C5-T1 roots	Neuropathic pain	[43]
	Meng et al.	2019	Rats	C5-T1 roots	Neuropathic pain	[44]
	Huo et al.	2020	Rats	C5-T1 roots	Neuropathic pain	[45]
	Wang et al.	2019	Rats	C5-T1 roots	Neuropathic pain	[15]
	Shen et al.	2019	Rats	C5-T1 roots	Neuropathic pain	[46]
	Fang et al.	2017	Rats	C5-T1 roots	SC motor neuron protection	[47]
Closed avulsion approach	Spinner et al.	2000	Rats	N. S.	Model establishment	[48]
	Yang et al.	2015	Rats	N. S.	Model establishment	[49]

N. S. refers to the idea that there is no specific root avulsion statement or the ratio of different types of avulsion roots was analyzed in the articles. SC: spinal cord.

## Data Availability

All data used in this study are available from the corresponding authors by request at “congrui@fmmu.edu.cn” and “luoceng@fmmu.edu.cn.”

## Abbreviations

BPI: Brachial plexus injury
OBPP: Obstetric brachial plexus palsy
DRG: Dorsal root ganglion
BPA: Brachial plexus avulsion
DREZ: Dorsal root entry zone
PKG-1: Cyclic GMP-dependent protein kinase-I
CCL2: C-C motif ligand 2
CCR2: C-C chemokine receptor type 2
TRPC: Canonical transient receptor potential.
